# Mapping of the Language Cortex

**DOI:** 10.7759/cureus.14960

**Published:** 2021-05-11

**Authors:** Faisal R Jahangiri, Gurtegh S Chima, Martha Pearson, Jacob Jackson, Arshad A Siddiqui

**Affiliations:** 1 Neurophysiology, Axis Neuromonitoring LLC, Richardson, USA; 2 Neurophysiology, Global Innervation LLC, Dallas, USA; 3 Neuroscience, School of Behavioral and Brain Sciences, The University of Texas at Dallas, Richardson, USA; 4 Neurosurgery, Neuroscience Institute, Hamad Medical Corporation, Doha, QAT

**Keywords:** language mapping, broca’s, wernicke’s, tumors, eloquent area, ionm, neuromonitoring, neurophysiology, cortical mapping

## Abstract

Awake craniotomy with intraoperative neurophysiological language mapping (INLM) is an established procedure for patients undergoing surgery to resection tumors in the language cortex area. INLM and continuous neurophysiological monitoring allow assessment of the language function, which is not possible under general anesthesia. INLM of the brain areas provides a helpful tool to the operating surgeon in reducing the risks associated with tumor resection in the motor and language cortex. We present a literature review and the technical method used for INLM by utilizing direct electrical cortical stimulation. We also report the usefulness of INLM for evaluation of the language function during resection of cortical tumors, epilepsy foci, and arteriovenous malformations (AVMs) located near language areas. First, the central sulcus is identified by sensory mapping, followed by the motor cortex's identification by direct electrical cortical stimulation (DECS). Neurological assessment of the patient is done by auditory and visual feedback. The patient is asked to repeat numbers, days, words, sentences, read words, and name pictures during cortical stimulation. DECS may cause a slurring or speech arrest. Electrocorticography (ECoG) is also performed during cortical stimulation to identify any after-discharges. Examination of the patient occurs immediately after surgery, and then 24 hours, one week, six months, and 12 months postoperatively. Bipolar DECS for motor mapping with ECoG can safely and reliably be utilized to identify essential language areas with minimizing permanent language deficits and maximizing the extent of tumor resection.

## Introduction

A neurosurgical procedure such as an awake craniotomy for resection of epileptic foci or gliomas is safely used near the brain's language areas. In the 1860s, the language areas of the brain were identified by Broca and Wernicke. Later in 1886, Sir Victor Horsley identified an epileptic focus by electrical stimulation of the cortex in an awake surgery. In the 1940s-50s, an American-Canadian neurosurgeon Wilder G. Penfield performed and published mapping of the brain with electrical stimulation response during awake epilepsy surgery [1]. The neurolept anesthesia (NLA) was introduced in the 1960s, which allowed surgery in awake patients without any tracheal intubation. After the introduction of intra­venous anesthesia using propofol in the 1990s, awake craniotomy with functional brain mapping with electrical stimulation became more common [1].

The mapping of the language areas helps in preventing permanent postoperative dysfunction by preserving these language areas. Presently, researchers are attempting to fully explore and standardize the areas responsible for language-eloquence to decrease the likelihood of injury during tumor removal and epilepsy surgeries. Emerging technologies such as functional imaging of the brain and intraoperative neuronavigation and functional brain mapping with electrical stimulation under an awake state may facilitate a more significant expansion of resection area while feasibly minimizing risk and the associated morbidity profile [1].

Surgical resection near the cortical language area involves a risk of permanent changes to the recovering patients' lifestyle. Long-term deficits in verbal/linguistic ability and perceptual lifestyle changes can be observed, where social exchange behavior is greatly limited by the intended but unguided surgical procedure.

## Technical report

Preoperative assessment

Functional magnetic resonance imaging (fMRI) can help identify various brain areas by activating them during a particular mental process. Diffusion tensor imaging (DTI) can identify the white matter fibers traveling as bundles or tracts (tractography). In 1948, Jun Wada developed the Wada test, which is considered the gold standard test for identifying the cerebral hemisphere dominant for the language area. Wada test was considered as such but due to its invasiveness and its limitations in identifying the language area because the ability to suppress hippocampal function with intracarotid infusion is not always consistent because much of the blood supply can be from the posterior circulation.

A day before or on the day of the surgery, preoperative language testing of the patient is performed to identify the patient's baseline language status and used as a reference during the surgery through language mapping (Tables 1, 2). Preoperative language testing can be primarily to familiarize the patient with the test style. This mitigates any discrepancies in the patients' performance during the surgery, limiting the possible occurrence of an error during the intraoperative awake testing without appropriate stimulation of the cortex's areas. Test battery must be given before the surgery, before anesthesia, and then once again, during the surgery (Table 3).

**Table 1:** Broca's area Broca's language area, its sub-regions, function, associated deficits, tasks used to identify aphasias, potential significant events, and possible errors during language mapping [1].

Language Region	Sub-Region	Function	Associated Deficits/Aphasias	Tasks used to identify aphasias	Possible significant events	Possible error (false localizations)
Broca's Area (BA)	Pars Opercularis	Speech production, commonly used preposition & word generation	Speech arrest, failure to produce words, hesitation, slurring	Rote tasks (counting, alphabet, days of the week, months of the year), preposition task	Arrest, failure to produce words, hesitation slurring, incorrect/nonsense words, perseveration	Activation of face motor area interpreted as BA
	Pars Triangularis	Speech production	Loss/difficulty producing language	Rote tasks (counting, alphabet, days of the week, months of the year), noun & verb production task	Slowing, slurring, hesitation, arrest	Activation of face motor area interpreted as BA, missing hints, word category errors
	Pre-Frontal Cortex (Pars Orbitalis)	Word production & development (nouns/verbs)	Inability to produce language, failure to carry complex & simple conversation	Repeating (words, sentences), noun & verb production task	Slowing, slurring, hesitation, arrest, literal & verbal paraphrasing errors, nonsense words	Activation of face motor area interpreted as BA

**Table 2:** Wernicke's area (WA) Wernicke's language area, its sub-regions, function, associated deficits, tasks used to identify aphasias, possible significant events, and potential errors during language mapping [1].

Language Region	Sub-Region	Function	Associated Deficits/Aphasias	Tasks used to identify aphasias	Possible significant events	Possible error (false localizations)
Wernicke's Area (WA)	Superior Temporal Gyrus (STG)	Language comprehension (with primary auditory input)	Loss/difficulty understanding language, anomia	Sentence Completion (SC), Phrase Repetition (PR), ON	Error in the selection, patient not understanding, need a question repeated, random word phrase generation, literal/verbal aphasia	Passing off a subtle language event as an environmental issue (patient having trouble hearing)
	Angular Gyrus (AG)	Receives visual input related to language comprehension/reading	Primarily visual-based language deficits (reading)	Word/sentence reading, ON in adjunct with the above	The patient having trouble visualizing words/sentences, literal/verbal aphasia, paraphasias, hesitation	Passing off a subtle language event as an environmental issue (assuming problems are a result of anesthesia/exhaustion)
	Supramarginal Gyrus (SMG)	Process visual/auditory receptive language projects to frontal language areas	Difficulty/inability to comprehend speech, including morphological errors, literal/verbal paraphasic errors. Neologisms, acalculia	DECS disruption	N/A	Misrepresenting visual language deficits as an inability to see presentation stimuli
Inferior Temporal Language Area (ITLA)	N/A	Primarily ON	Anomia	ON	Inability to name objects	Inability to name objects
Arcuate Fibers	N/A	Linking WA with BA language areas	Conductive aphasia, fragmented/broken speech, inarticulate	Combination of Repetition, word generation, SC	N/A	N/A

**Table 3:** Language mapping Various tasks for language testing [1].

Language Tasks used for localization		
Test Name	Test Description	Test Examples
Rote Language Task (RLT)	Typically used for Broca's area aphasias, a spontaneous speech production task.	Counting from 1 to 20 Serial's 2 tasks, 'Count from 1-20 by 2s' Naming days of the week or months of the year Naming letters of the alphabet from a specific letter
Noun/Verb Word Production Task (CNVWP)	More complex auditory input typically testing Broca's area aphasias, Specific for production of nouns/verbs when the comprehension of the presented sentence is intact.	Noun task List three fruits Name three pizza toppings Verb Task What do you do in a pool? What do you do in a car?
Phrase Repetition Task (PR)	Usually used for Broca's area aphasias characterized by repeating specific phrases	Yesterday was Monday. He likes vanilla ice cream.
Object Naming Task (ON)	Utilized for Broca's area, Wernicke's area and the Angular Gyrus, a visual input nomic task	Identifying objects from flashcards Identifying actions from flashcards
Sentence Completion Task (SC)	Typically used to test for Wernicke's area aphasias, an auditory input comprehension task	A pilot flies a _____. An ATM dispenses _____. Brad Pitt is an _____.
Question and Answer Task (QA)	Usually used to test for Wernicke's area aphasias, comprehension test requiring response	Where are you? How many hours are in a day? Where is Germany?
True and False Task (TF)	Usually used to test for Wernicke's area aphasias, comprehension test requiring response	Donkeys can breathe under water. You use a pen to write a letter. The president is an alien from Mars.
Sentence Reading Task (SR)	Visual-based input modality normally used to test for Wernicke's area aphasias	Reading simple sentences, 'The dog ate his bone." Reading complex sentences, 'Mac, while at the grocery store, received a call from his wife to remind him to purchase apples."

Anesthesia

The asleep-awake-asleep cycle is used for craniotomy procedures with language mapping [2,3]. The patient is intubated with total intravenous anesthesia (TIVA) using propofol (or sodium pentothal). A Mayfield head frame stabilizes the patient's head with implanted pins. Hyperventilation is performed before opening the dura. The patient is extubated and awakened for mapping of the language areas. Once the mapping and the surgical procedure are complete, the patient is reintubated with a fiberoptic laryngoscope for general anesthesia.

Alternately, during the conscious sedation protocol, the sedation is initiated and maintained with propofol or midazolam and/or dexmedetomidine. Fentanyl or sufentanil is used for analgesia. The infusion is typically stopped 10-15 minutes before the mapping. The patient is awake for mapping during the critical parts of resection during the surgery. Once the resection is complete, the propofol is started again.

Intraoperative neurophysiological language mapping (INLM)

After patient sedation, the patient is positioned in the supine position with his head affixed to the three-pin Mayfield frame. The stimulation surface adhesive electrodes for median nerve somatosensory evoked potentials (SSEP) are placed on the wrists bilaterally. Subdermal needle electrodes for electromyography (EMG) recordings are placed in the contralateral face, upper, and lower extremity muscles. Subdermal needle electrodes are also placed on the scalp for SSEP and electroencephalogram (EEG) recordings. Baseline median nerve SSEP responses are recorded bilaterally along with the baseline scalp EEG [4].

The stereotactic navigational system is then registered and used to make an appropriate skin incision. The head is prepped using betadine and alcohol solutions. A scalp block is done using a local anesthetic injected into the dermis. A skin incision is made. The skull surface overlying the tumor is exposed, and the stereotactic system is used to identify the tumor margins. A standard craniotomy is performed.

Awake craniotomies are used frequently in contemporary neurosurgery due to low failure rates, with failure constituting both expressive and perceptive linguistic limitations in the patient postoperatively. While various protocols exist to map language areas during these operations, we will outline the process to provide a guide for effective operational analysis of linguistic centers for surgical excision of brain lesions. While the patient is awake, the test battery is again repeated, with the surgeon using the following procedure for testing the cortex and simultaneously referencing the patient's task performance (Table 3).

Sensory Mapping

**Figure 1:**
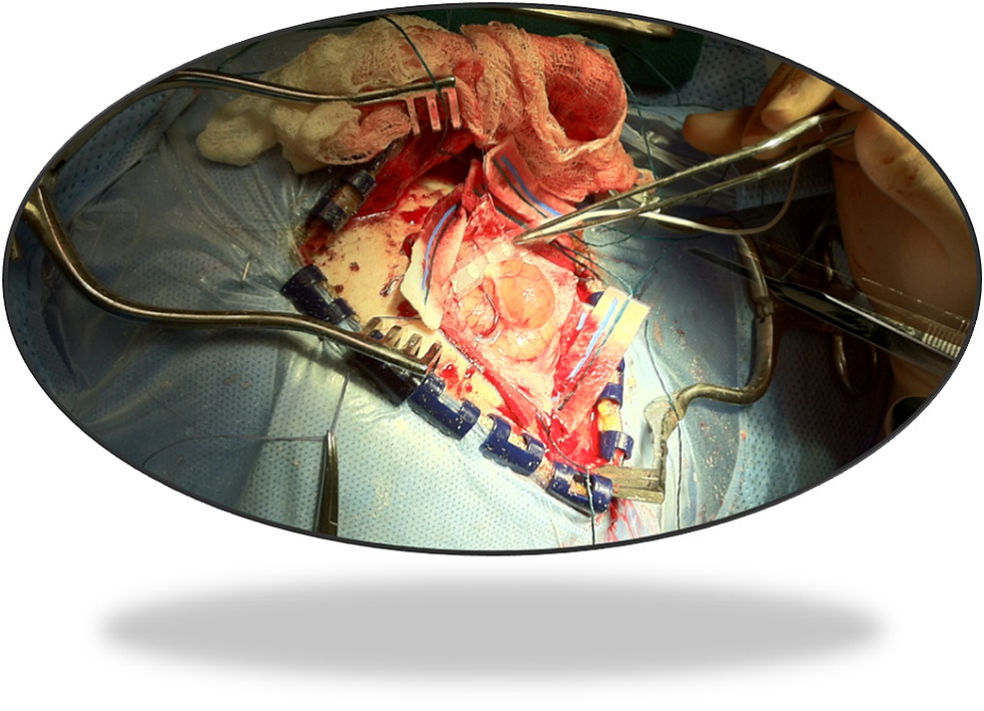
Cortical grid placement over the exposed brain Placement of a cortical grid (six contact 1 x 6) over the exposed area for sensory mapping phase reversal.

**Figure 2:**
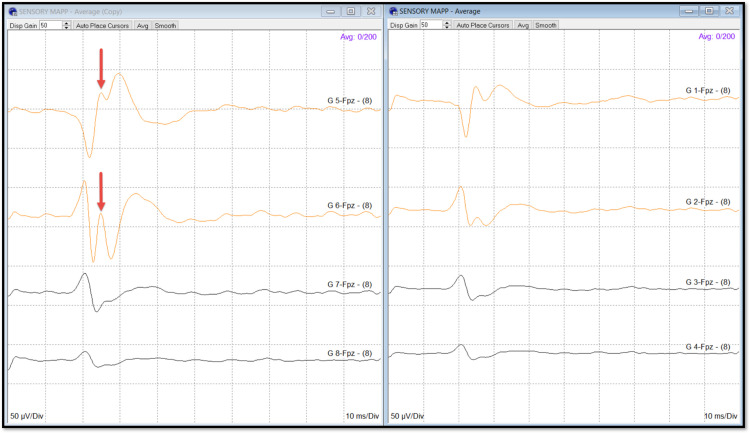
Cortical sensory mapping Sensory mapping phase reversal. On-axis median nerve sensory mapping by a 2 x 4 grid with a triphasic phase reversal, including a P25 response. The phase reversal is between G1/2 and G5/6. The responses from G1 and G5 are pre-central, and G2, G3, G4, G6, G7, and G8 are post-central.

Following adequate exposure of the cortex, a six-grid (1 x 6) or eight-grid (1 x 8 or 2 x 4) subdural electrode is placed on the cortical surface over the area of the presumed central sulcus (CS) for waveform interpretation in all four planes, anterior-posterior and medial-lateral. Contralateral median nerve stimulation is used to generate phase reversal at the central sulcus (Figure 1). Recording filters are set at 30-3000 Hz with a time base of 100 milliseconds [5]. The CS and somatosensory cortex are localized by generating multiple sensory maps by stimulating the contralateral median nerve at a current of 20-35 mA between frequencies of 2.66-4.79 Hz. Mapping continued until the triphasic waveform was generated with a P25 peak directly over the central sulcus, a part of the P20/N30-N20/P30 complex which is either over the pre- or post-central sulcus area (Figure 2).

Craniotomy should expose a tumor (or implicated epileptogenic tissues) and 3 cm of the surrounding brain surface. A hand-held bipolar stimulation probe with 1 mm diameter contacts positioned 5 mm apart is used for direct cortical stimulation. A biphasic square wave of 200-500 microseconds (µs) pulse width, trains of 50 or 60 Hertz (Hz), starting with a low stimulus constant current at 1.5-mA and increased to a maximum of 20 mA or identification of after discharges (ADs). However, a square-wave pulse under 7.0 mA is preferred. The cortex is mapped every 5-10 mm, and positive stimulation sites at which language impairment was caused (by the process of awake craniotomy testing) are marked with sterile numbered tickets [1]. The cortex is then excised/lesioned with minimal damage done to cortical tissues responsible for successful communication when deep anesthesia is resumed. The surgery can then conclude with the surgeon completing the procedure.

Motor Mapping (By Direct Electrical Cortical Stimulation - DECS)

**Figure 3:**
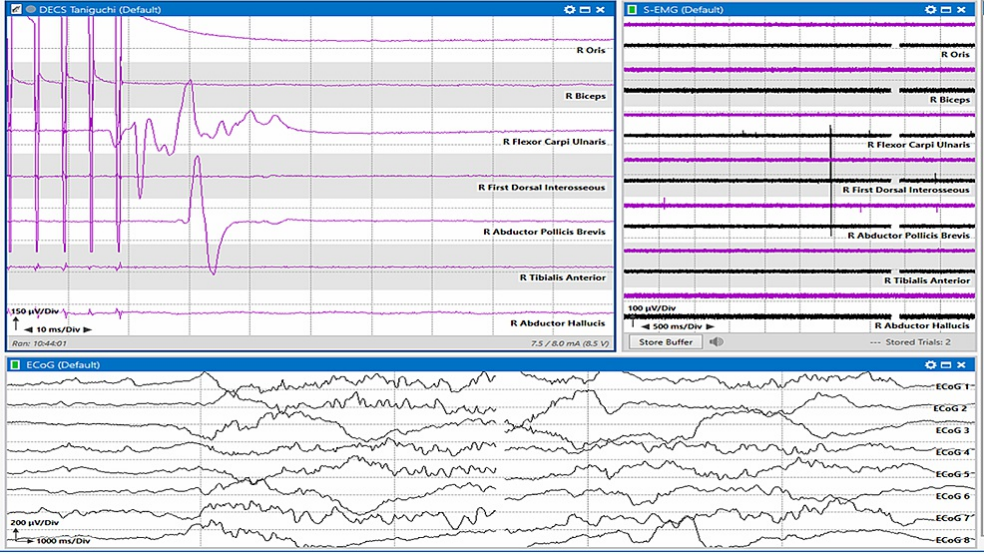
Taniguchi method High-frequency motor mapping. Top left: motor mapping responses from right flexor carpi ulnaris and abductor pollicis brevis muscles. Top right: spontaneous electromyogram (EMG) recording from the contralateral muscles. Bottom: Electrocorticography (ECoG) recordings from a 1 x 8 grid.

**Figure 4:**
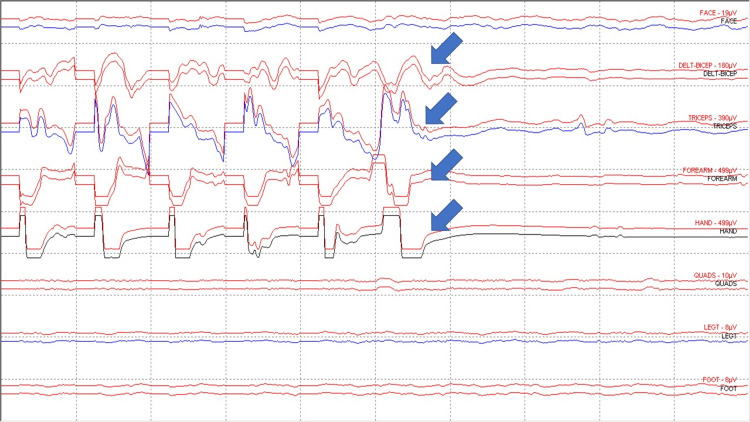
Penfield method Slow frequency motor mapping. Motor mapping responses from deltoid-biceps, triceps, and forearm (flexor carpi ulnaris and brachioradialis) and hand (abductor pollicis brevis-abductor digiti minimi muscles (blue arrows).

**Figure 5:**
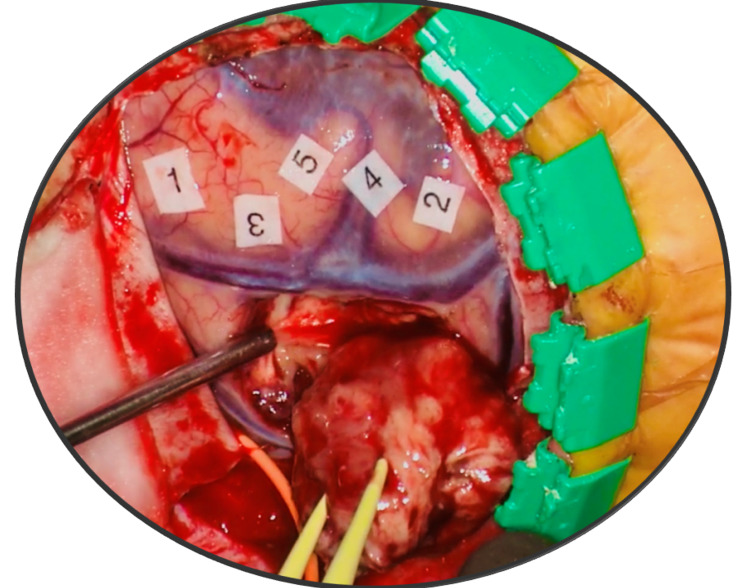
Motor cortex After localization and verification of the exact location of the motor areas, the tumor was resected. The patient underwent continuous awake testing of the right upper and lower extremity motor function. Papers marked with 1, 2, 3, 4, and 5 represent various cortical functional areas.

After localization of the central sulcus, motor mapping of the cortex is performed by using direct electrical cortical stimulation (DECS) and electrocorticography (ECoG). DECS can be done by two methods [6]: (A) The high-frequency short pulse Taniguchi method is performed by using a monopolar anodal hand-held ball tip probe with a pulse duration of 500 µs, 4 or 5 pulses at a frequency of 250-500 Hz. The stimulation intensity is applied between 2-30 mA. (B) The slow frequency long pulse Penfield method is performed by using hand-held bipolar ball tips probe with a pulse duration of 300-500 microseconds at a frequency of 50/60 Hz. Stimulation is applied for three to five seconds at each site. During the stimulus, the subdural grid electrodes are placed on the exposed cortex close to the stimulation sites to monitor ECoG for the presence of ADs or seizure activity (Tables 4, 5). The stimulation is initiated at an intensity of 2.0 mA and increased as needed until a positive response (compound muscle action potential- CAMP) or after discharges (ADs) is elicited, or until clinical seizure activity is encountered (with a maximum limit of 20 milliamperes/mA) (Figures 3-5) [6].

**Table 4:** Stimulation parameters Penfield and Taniguchi's methods suggested stimulation parameters. Hertz = Hz, microseconds = µs, milliseconds = ms, milliamperes = mA, seconds = s.

Stimulation Parameters		
Specification	Penfield	Taniguchi
Type of Stimulator	Bipolar	Monopolar
Type of Pulse (Phase)	Biphasic or Monophasic	Monophasic
Frequency	50/60 Hz	250-500 Hz
Pulse width	300 – 1000 µs	500 µs
Intensity	2 – 10 mA	2 – 20 mA
Duration of stimulation	2 – 5 s	10 – 20 ms

**Table 5:** Recording parameters Recording parameters suggested by Penfield and Taniguchi. Hertz = Hz, microvolts = µV, milliseconds = ms, division = div, Electrocorticography = ECoG.

Recording Parameters		
Specification	Penfield	Taniguchi
Low-cut filter	10 Hz	10 Hz
High-cut filter	5000 Hz	5000 Hz
Notch Filter	Off for ECoG	Off for ECoG
Gain	200-500	200-500
Sensitivity	200 µV	200 µV
Time-base	100 ms/div	10 ms/div

There are two types of responses to electrical stimulation of the brain. When the response is induced, it is known as a "positive mapping," when the response cannot be induced, it is known as "negative mapping."

Language Mapping (By DECS)

**Figure 6:**
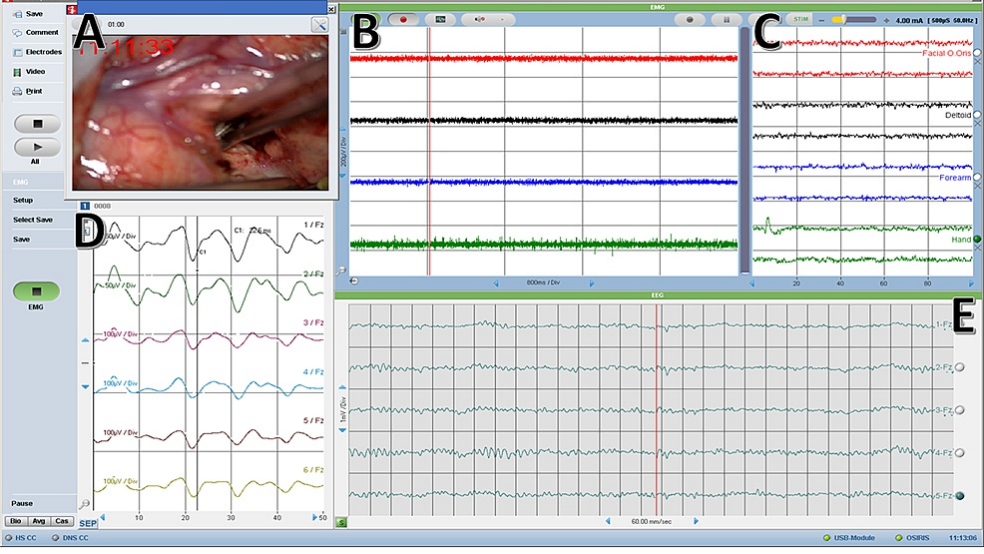
Intraoperative neurophysiological language mapping (INLM) Intraoperative recordings during a language mapping awake craniotomy. (A) Video input from the microscope showing the cortex. (B) Spontaneous electromyogram (EMG). (C) DECS - Direct electrical cortical stimulation. (D) Median nerve phase reversal (PR) window. (E) Electrocorticography - ECoG.

After localization of the central sulcus and motor mapping, language mapping is performed by DECS and ECoG. DECS is performed by the Penfield method using hand-held bipolar ball tips probe with a monophasic or biphasic pulse duration of 0.5-1.0 milliseconds (ms) at a frequency of 50/60 Hz. The stimulation is applied for three to five seconds at each site. During stimulation, the subdural grid electrodes are positioned close to the stimulation sites to monitor ECoG for the presence of ADs or seizure activity. The stimulation is initiated at an intensity of 2.0 mA and increased as needed until a positive response (speech arrest/slurring) or after discharges (ADs) were elicited or until clinical seizure activity was encountered (with a maximum limit of 15 mA for biphasic stimulation) (Figure 6). If ADs are present after DECS, ice saline (4˚C) must be applied immediately to the exposed cortical surface to stop the ictal activity [7].

**Figure 7:**
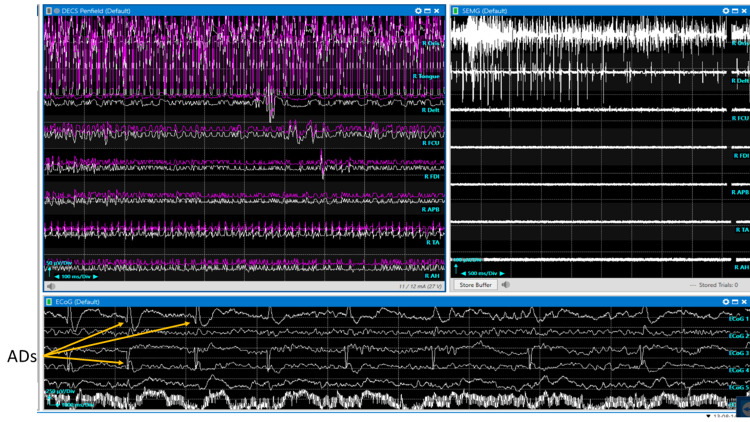
Intraoperative neurophysiological language mapping (INLM) Language mapping. Intraoperative recordings during a language mapping awake craniotomy. (Left upper) Direct electrical cortical stimulation (DECS) with responses from orbicularis oris and tongue muscles. (Right upper) Spontaneous electromyogram (EMG). (Bottom) Electrocorticography (ECoG) with after discharges (ADs) present after direct cortical motor stimulation (orange arrows).

For each stimulated site, the patient is tested for various language tasks to evaluate brain function. The tests include reading sentences, auditory comprehension, visual comprehension, and spontaneous speech (Table 5). A positive mapping is a stimulation-induced language disruption in the absence of ADs. If there is a lack of stimulation-induced language disruption, it will be labeled as negative mapping. The frontal language sites are more predictable, and the temporal sites are variable. The language areas can be successfully identified in patients with gliomas. Language tasks such as number counting, object naming, and reading are performed during language mapping of the frontal and temporal lobes (Figure 7). The cortical areas are identified as a positive mapping if the patient cannot count, repeat words, read words, or name objects in two out of three stimulations. Speech arrest can be the result of language function disturbance and motor function disturbance. All cortical areas with positive mapping, vasculature, and subcortical white matter tracts must be preserved during resections [8].

The resection is then performed in these patients, repeating the same task, and a margin of 1 cm around the identified language areas is left. The resection of the cerebral tumors is performed if they show no language task response during electrical stimulation. The subcortical motor mapping can also be achieved by monopolar cathodal stimulation or bipolar stimulation.

There are some challenges when performing language mapping in awake patients. If the patient has a soft voice, it is recommended to use a microphone to amplify the sound volume. The sedation should be reduced if the patient is falling asleep during the mapping part. If the patient is getting tired, start the mapping from the most critical part. A soaked swab can help the patient if the patient complains about a dry mouth.

## Discussion

The focus of neurology was once restricted to the conception of a holistic error within the brain tissue (encompassing the entire brain) or the behavior of a person being entirely separate from the brain's functioning. In combining his research on epilepsies and disconnection syndromes with his readings of Wernicke, Geschwind found a complex and multilayered explanation for aphasias that implicated lesions located in association pathways; when severe, these would cause behavioral disorders such as aphasias. The behavioral evaluation is performed during awake craniotomy procedures [8]. It effectively determines the functional ability to retain/possess linguistic functioning before, during, and after a procedure. This intraoperative behavioral analysis minimizes unknowing lesioning of critical language areas through neural/behavioral monitoring during the resection.

Since the 1930s, surgeons and researchers could stimulate the brain directly. In recent years, intraoperative cortical stimulation has been adopted to identify and preserve language function and motor pathways during surgery. We have a limited understanding of the mechanism of stimulation effects on language. The principle is based upon local neurons' depolarization and passing pathways, local excitation or inhibition, and possible orthodromic or antidromic propagation to more distant areas. Such stimulation might be performed to determine the focus of epileptic seizures in a specific patient but can be used for linguistic cortex mapping.

Language mapping techniques were initially developed in the context of operations for treating epilepsy surgically. Large-scale craniotomies expose the brain well beyond the region of surgical interest to localize multiple cortical regions containing stimulation-induced language areas. These are termed "positive sites." Positive sites consist of neurons that respond to a small electric voltage with spreading cortical activation and linguistic disruption during analyses (observed during behavioral reading/reciting tasks). Researchers have been able to identify positive language sites in 95-100% of a given experimental sample [8]. Negative mapping helps in directing resection of local cortical regions, containing no response to direct cortical stimulation applied to known language-related areas. Negative mapping becomes relevant in the absence of identified positive sites. By using negative sites, surgeons can reduce the cortical exposure necessary, increasing patient safety and efficiency.

Classical anatomical standards are insufficient to predict the location of language areas accurately. Variability of localization among individuals is one of the most consistent literature findings [8]). Therefore, the boundaries of language areas are not well defined. Language-associated activity has even been found in the primary motor cortex and the Sylvian fissure. There are interlobar differences in cortical excitability [3]. The temporal language area threshold is 1.5 times higher than the threshold of the frontal language cortex. If there is a lesion near the language cortex, the threshold is increased 2.6 times. The edema in the language cortex's proximity increases its threshold by 1.8 times [9].

It is not possible to find the positive language sites before resection. Despite negative brain mapping, permanent postoperative neurologic deficits have been reported. This suggests the identification of negative sites may not be sufficient for limiting postoperative deficits. For example, a study of patients who underwent a glioma resection showed the patients with permanent postoperative neurologic deficits neglected to have had identified positive sites [8]. This information would lend itself to a conclusion purporting the necessity of mapping for positive sites and negative sites.

In the recent literature, approximately 90 publications examine the utility of intraoperative stimulation mapping techniques in achieving a greater extent of resection for gliomas while minimizing morbidity. Within these studies, cohorts varied between 20 and 648 patients, with a median of 50 patients per study. Nearly all the reports provide level III evidence supporting microsurgical adjunct, except for two randomized studies that examined anesthetic and fluorescence-guided techniques to maximize the extent of resection [8]. Whereas pre- and intraoperative mapping and monitoring of motor and language function have already been established, the possibilities of neuropsychological or cognitive mapping and monitoring should be investigated more intensely. Neuropsychological testing before, during, and after glioma surgery should always be performed [10-12]. If the distance of the resection margin from the nearest language site is >1 cm, significantly fewer permanent language deficits will occur [13].

Currently, glioma resection operations using intraoperative neural monitoring have provided the most positive evidence for the technologies. Surgeons have reported the surgeries as having been more efficient, certainly more effective, and prolonged survival rates. Motor evoked potential recordings during surgery have been evidenced to prevent maladaptive cortical changes and increase tumor resection percentages.

## Conclusions

Intraoperative language mapping with cortical and subcortical stimulation is a reliable method for awake glioma resection surgery near functional pathways. In some patients, it may be beneficial to stimulate at a higher intensity at each cortical site regardless of the adjacent cortical areas' threshold. Resection with language mapping is associated with minimal postoperative neurologic deficits in language and speech functions. Determination of the improvement in preoperative language deficits and the duration of the postoperative deficits depends on the distance of the resection margin from the language sites. Cortical stimulation mapping for identifying essential language sites in patients with gliomas of the dominant hemisphere temporal lobe will maximize the extent of tumor resection and minimize permanent language deficits.
